# The effects of inbreeding on covering success, gestation length and foal sex ratio in Australian thoroughbred horses

**DOI:** 10.1186/s12863-020-00847-1

**Published:** 2020-04-08

**Authors:** Evelyn T. Todd, Natasha A. Hamilton, Brandon D. Velie, Peter C. Thomson

**Affiliations:** 1 School of Life and Environmental Sciences, The University of Sydney, Sydney, NSW 2006 Australia; 2 Racing Australia Equine Genetics Research Centre, Racing Australia, Sydney, NSW 2000 Australia

**Keywords:** Inbreeding, Genetic diversity, Thoroughbred horse, Fertility

## Abstract

**Background:**

Horses produce only one foal from an eleven-month gestation period, making the maintenance of high reproductive rates essential. Genetic bottlenecks and inbreeding can increase the frequency of deleterious variants, resulting in reduced reproductive levels in a population. In this study we examined the influence of inbreeding levels on foaling rate, gestation length and secondary sex ratio in Australian Thoroughbred mares. We also investigated the genetic change in these traits throughout the history of the breed. Phenotypic data were obtained from 27,262 breeding records of Thoroughbred mares provided by three Australian stud farms. Inbreeding was estimated using the pedigree of each individual dating back to the foundation of the breed in the eighteenth century.

**Results:**

While both gestation length and foaling rate were heritable, no measurable effect of inbreeding on either trait was found. However, we did find that the genetic value for both traits had decreased within recent generations. A number of environmental factors also had significant effects on foaling rate and gestation length. Secondary sex ratio had only an extremely small paternal heritable effect and was not susceptible to environmental influences.

**Conclusions:**

In contrast to racing performance, inbreeding had no measurable effect on foaling rate or gestation length in Australian Thoroughbred horses. This could be because the level of inbreeding in the population examined is not high enough to show a discernible effect on reproductive traits. Populations that experience higher levels of inbreeding due to use of artificial reproductive technologies or extremely small population sizes may show a more pronounced reduction in natural foaling rate or gestation length. It is also possible that the intensive management techniques used in the Thoroughbred population masks any negative effects of inbreeding. The decrease in the genetic value of foaling rate is likely to be because horses with unfavourable genetic potential have not yet been selected out of the population. The change in genetic value of gestation length may be due to selective breeding favouring horses with shorter pregnancies. We also found that prioritising the mating of older mares, and avoiding out of season mating could lead to an increased breeding success.

## Background

Horses have an 11 month gestation period, both conceiving and giving birth in the spring and summer [1]. Like most spring seasonal-breeding animals, increased photoperiod induces ovulation cycles in mares. Survival of twins is rare, so mares can only produce one foal a year. The long gestation period and short breeding season make the maintenance of good fertility rates in horse populations imperative to provide commercial returns for domestic breeds, and to increase the size of endangered populations [2, 3].

Deleterious genetic variants have accumulated in the genomes of modern horses as a result of population bottlenecks during domestication and breed foundation events [4, 5]. In this process, also known as the “cost of domestication”, deleterious mutations increase in frequency by “hitchhiking” on selective sweep regions [6]. These mutations can also increase in frequency through inbreeding, selective breeding and genetic drift in a population [7]. The presence of these variants in a population can have negative consequences for overall fitness, including a decrease in fertility rates. However, it is also possible that selective breeding over a number of generations may have removed some or all of these deleterious variants from contemporary horse populations. Evidence of positive selection in regions harbouring genes related to conceptus development have been found in domestic horse breeds [8, 9], indicating that fertility rates may have been targeted and improved by breeding practices.

The effects of inbreeding on reproductive traits vary between studies. Increased inbreeding levels were associated with reduced fertility in some domestic and wild horse populations [2, 3, 10]. Impaired ovarian function resulting from high levels of inbreeding was reported in the Przewalski’s horse, the most closely related species to the domestic horse [3]. Conversely, a number of studies in other horse breeds have shown no relationship between inbreeding levels and reproductive traits [11–13]. Varying relationships between inbreeding and reproductive performance also exist for a number of other domestic animal populations [14–18]. It is possible that the effects of inbreeding on fertility may vary between different populations depending on the rate of increase in inbreeding, selective pressures and genetic diversity.

As well as affecting fertility, there is some evidence that increased inbreeding can skew secondary sex ratios (the sex ratio at birth) in animal populations [19]. Variations in sex ratio exist due to an increased chance of early conceptus loss of one sex under different conditions [20]. As maternal condition declines due to environmental stresses or inbreeding, the chance of producing a viable male conceptus may also decrease [19, 21]. Early female horse conceptuses produce more insulin like growth factor-1 than males, which may promote their survival in adverse conditions [20]. It is possible that the environment at the time of conception or levels of inbreeding in horses may favour the survival of one sex.

Maintaining high reproductive rates is particularly important for the Thoroughbred horse breed. The Thoroughbred population has been closed since the eighteenth century, resulting in reduced levels of genetic diversity in current individuals [22–24]. Although selective breeding in Thoroughbred horses focusses on improving racetrack performance, the prohibition on reproductive technologies also makes it essential to maintain good fertility rates in the population. Thoroughbred foals that are born early in the spring season are assumed to have a size and maturing advantage over their peers. Mares that can conceive within 30 days after parturition will give birth at the same time next year. However, mares often take more than one covering each season, leading to their parturition date being delayed until later in the spring every year [25]. When a mare’s parturition date reaches the end of spring, she cannot be covered until the next year. This will result in a two-year period in which she does not produce a foal.

Despite many generations of selective breeding for athletic ability, increased inbreeding is associated with reduced racing performance in Thoroughbred horses [24]. In this study we examine the effects of inbreeding levels on foaling rate, gestation length and secondary sex ratio in Thoroughbred mares. We use the pedigree data of twenty-first century Thoroughbred horses to estimate the heritability and the effects of inbreeding on these three reproductive traits. We also evaluate the environmental effects on these reproductive traits in Australian Thoroughbred mares. Additionally, we used estimated breeding values to measure the genetic change in foaling rate and gestation length since the foundation of the Thoroughbred population in the eighteenth century. Estimated breeding values (or genetic values) can measure the genetic potential of an animal for a trait based on the phenotypic information of themselves and their relatives. Genetic values are not commonly used for Thoroughbred horses but are utilized to assist in breeding management for other horse and livestock breeds. Although phenotypic data are not available for previous generations, we utilize the comprehensive pedigree information available for Thoroughbred horses to calculate the genetic values for the ancestors in the pedigree, based on the reproductive trait data available for their modern day descendants.

## Results

Data were available for 27,296 coverings of 12,922 mares bred to 131 stallions between 2000 and 2017. The pedigree of these individuals dating back to the founders of the population consisted of 92,852 individuals. The conceptuses, mares and stallions in the dataset had an average pedigree depth of 29 generations and a mean level of inbreeding of 0.156 ± 0.012 (mean ± SD).

**Fig. 1:**
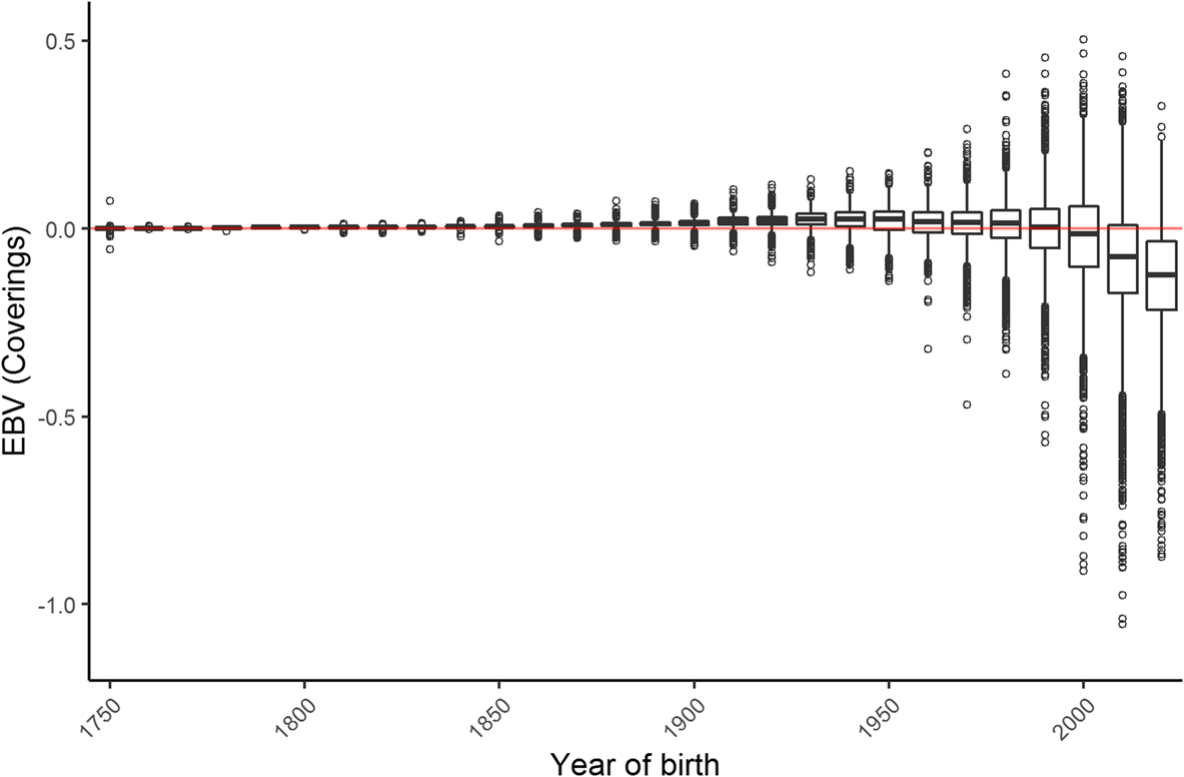
Boxplot of the distribution of estimated breeding values (EBVs) over time for Thoroughbred horses (*n* = 95,663), based on the foaling rate of 27,962 individuals bred between 2000 and 2017. Each bin represents a 10-year period

**Fig. 2:**
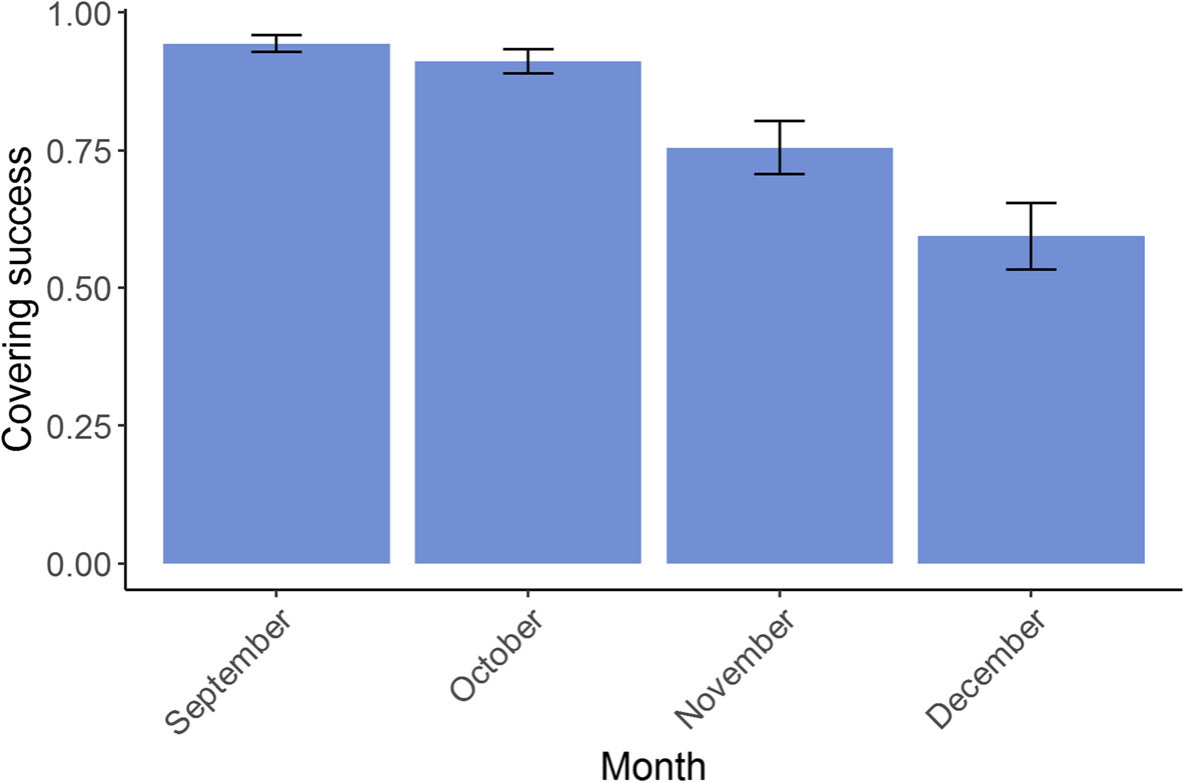
The relationship between the predicted values of foaling rate by month of covering for Australian Thoroughbred horses between 2000 and 2017 (*n* = 27,962). The error bars represents ±1 standard error of the predicted value

**Fig. 3:**
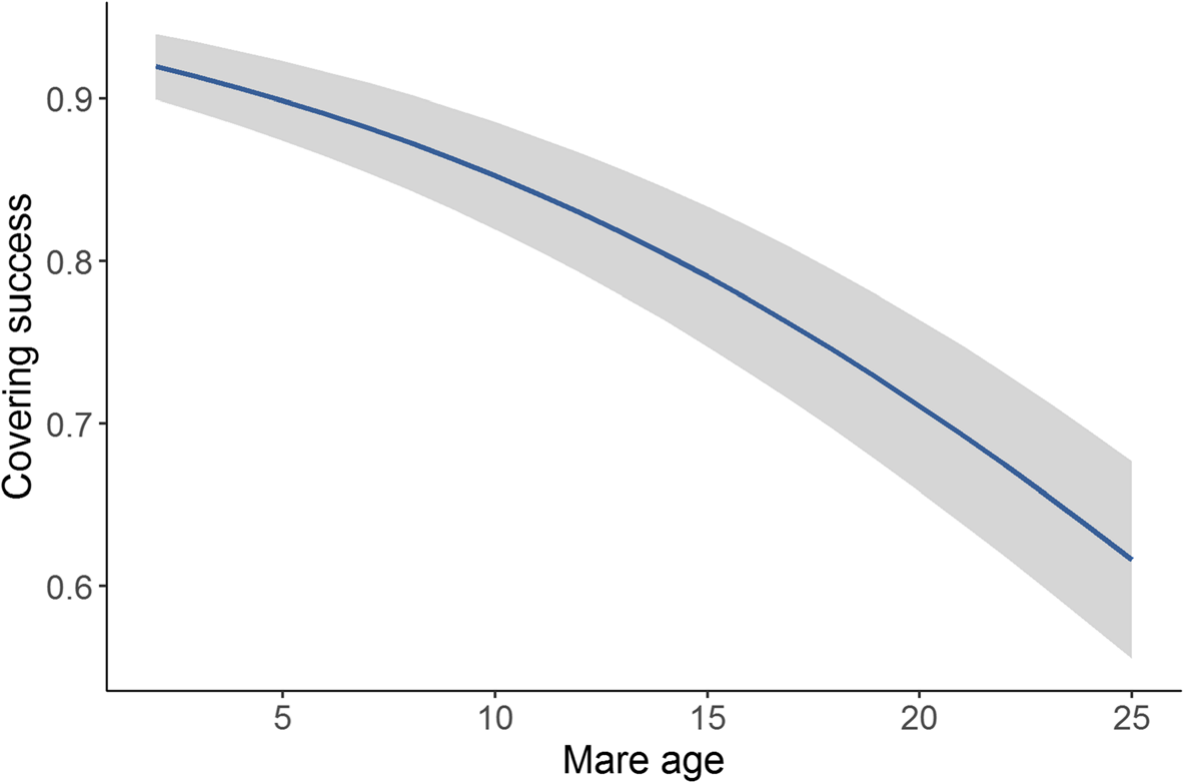
The relationship between the predicted values of foaling rate and mare age for Australian Thoroughbred horses between 2000 and 2017 (*n* = 27,962). The grey band represents ± standard error of the predicted value

The overall proportion of mares with a positive 15 day scan each season was 81.65% (22,287 out of 27,296). Based on the estimated variances from the mixed models, the maternal heritability of foaling rate was 0.058 (± 0.015), and 0.00 (± 0.00) for paternal heritability. Estimated breeding values for all individuals in the pedigree based on the foaling rate of their modern descendants showed a decrease in recent generations from a mean of − 0.002 (± 0.092) in 1990 to − 0.133 (± 0.138) by 2017 (Fig. 1). Variation in the estimated breeding values of foaling rate was also estimated to increase dramatically from 1990 onwards (between − 1.05 and 0.502) from previous years (between − 0.468 to 0.264) (Fig. 1). Mares that were covered later in the season showed a significant reduction in foaling success (*P* < 0.001) (Fig. 2). There was an overall decrease in foaling rate with increasing mare age (*P* < 0.001) (Fig. 3). Foaling rate had no relationship with the sire, dam or conceptus inbreeding level (*P* = 0.142, 0.788 and 0.701 respectively).

The sex of 7578 live foal births were recorded in the dataset, 3785 of which were colts (49.95%) and 3793 were fillies (50.05%). Secondary sex ratio did not have an estimated heritable component for maternal genetic estimates (0.005 (± 0.026)), but had a small paternal heritability estimate of 0.011 (± 0.005). The sex ratio was not influenced by the sire, dam or foal inbreeding level (*P* = 0.637, 0.746 and 0.899, respectively). Environmental variables of mare age and month of birth also had no significant relationship with sex ratio (*P* = 0.495 and 0.337, respectively).

**Fig. 4:**
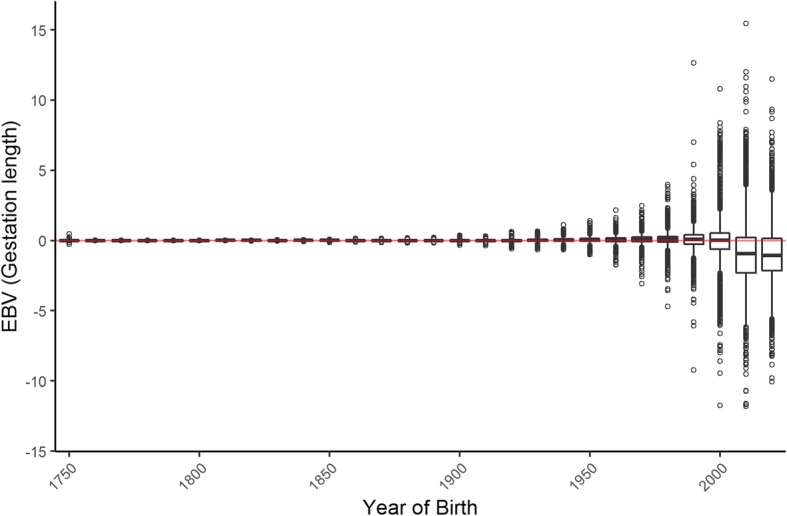
Boxplot of the distribution of estimated breeding values (EBVs) over time for Thoroughbred horses (*n* = 95,663), based on the gestation length of 764 individuals bred between 2000 and 2017. Each bin represents a 10-year period

**Fig. 5:**
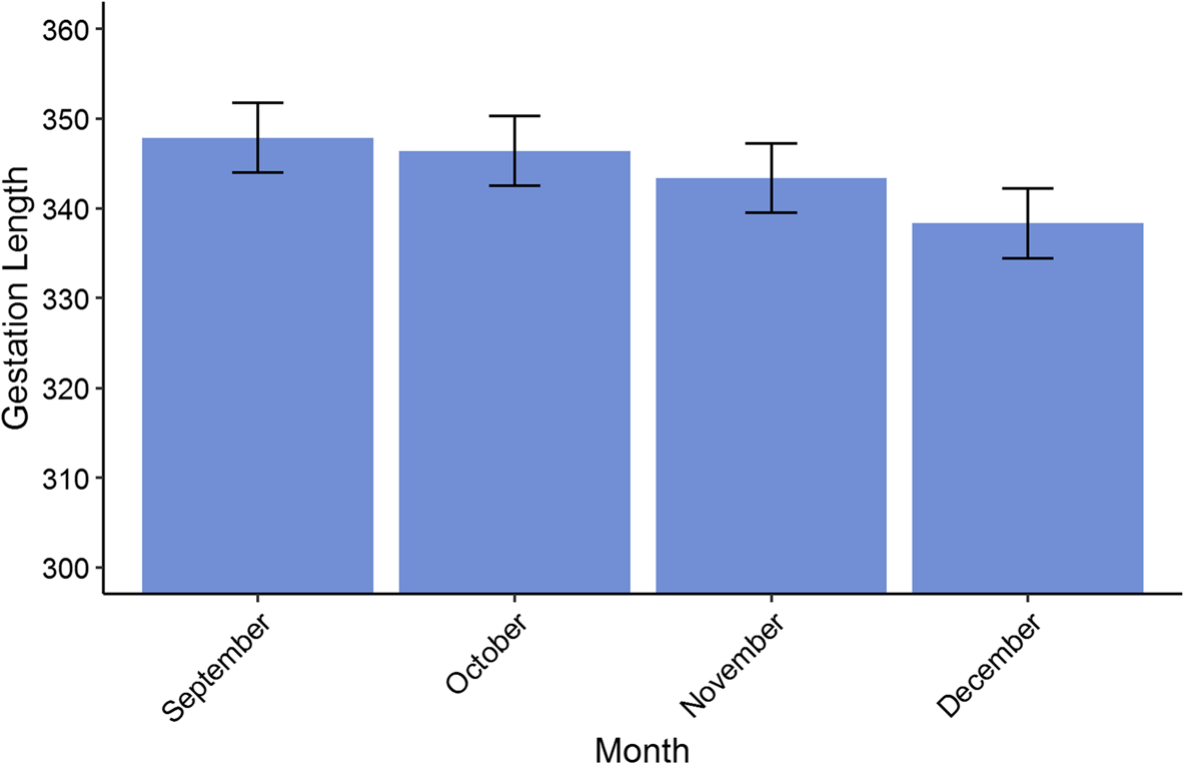
The relationship between the predicted values of gestation length by month of covering for Australian Thoroughbred horses between 2000 and 2017 (*n* = 764). The error bars represents ±1 standard error of the predicted value

**Fig. 6:**
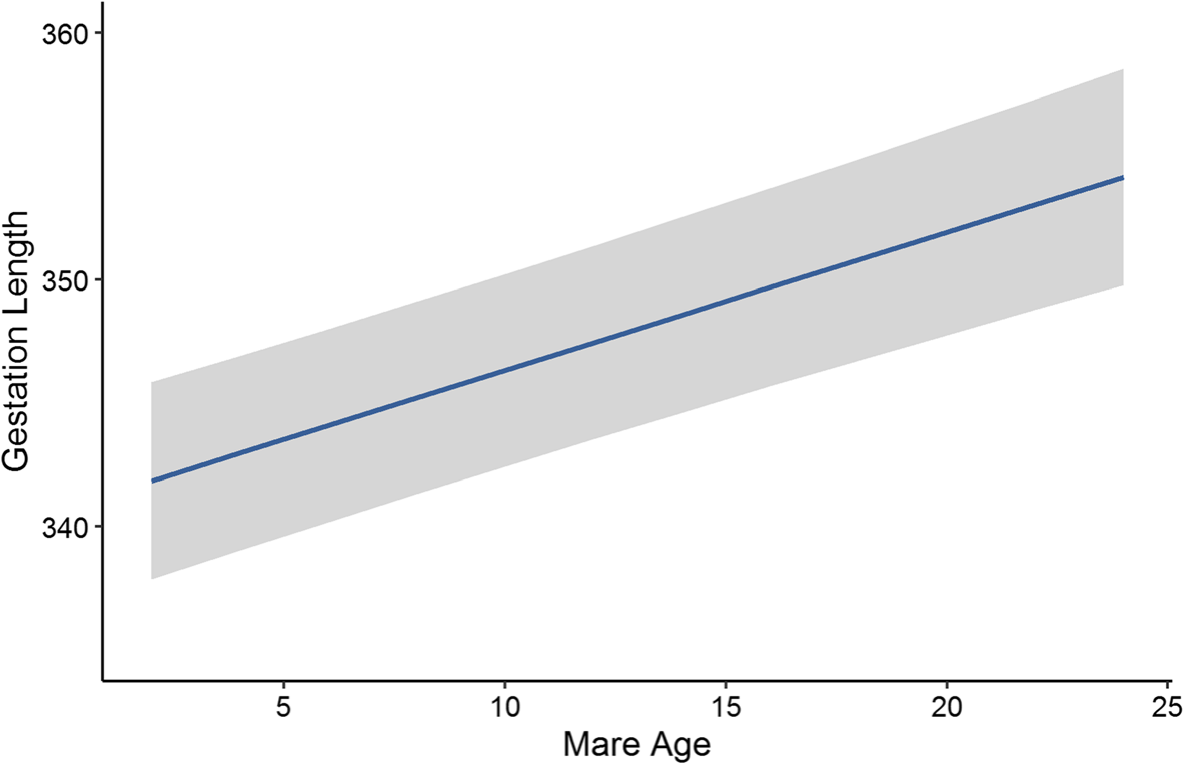
The relationship between the predicted values of gestation length by mare age for Australian Thoroughbred horses between 2000 and 2017 (*n* = 764). The grey band represents ± standard error of the predicted value

Gestation length data were available for 764 foals from 152 mares covered by 89 stallions. Gestation length was normally distributed, with a mean of 341 days (± 8.633), a minimum of 311 and a maximum of 376. Based on the estimated variances from the mixed model, the maternal heritability of gestation length was found to be 0.562 (± 0.042) and the paternal heritability was 0.004 (± 0.001). The average estimated breeding value in the pedigree remained at a mean of 0.03 (± 0.241) and showed little variation between − 4.695 and 3.959 from the foundation of the population until 1990 (Fig. 4). Since 2000, the average estimated breeding value for gestation length has decreased to − 0.7 (± 1.91) and variation has increased, with a minimum of − 11.82 and a maximum of 15.41. There was a significant decrease in gestation length in the later months of the season (*P* < 0.001) (Fig. 5). Gestation length increased linearly with mare age (*P* < 0.001), going from a mean of 342 days at 2 years old to a mean of over 354 days by 24 years old (Fig. 6). Male foals had a significantly longer predicted gestation length (349 days) than female foals (346 days) (*P* < 0.001). Gestation length had no significant association with the sire (*P* = 0.087), dam (*P* = 0.419) or foal (*P* = 0.062) inbreeding level.

## Discussion

We found that inbreeding had no measurable effect on foaling rate, sex ratio or gestation length in Australian Thoroughbred horses. This may be because intensive management techniques used on commercial Thoroughbred stud farms have masked any negative effects of inbreeding on these traits. Some studies have similarly shown that inbreeding has no effect on gestation length [12, 13], or foaling rate [11] in domestic horses. However, high levels of inbreeding in the endangered Przewalski and Sorraia horse breeds are associated with decreased birthing rates [2, 3]. It is possible that inbreeding has no measurable effects on reproductive traits until it reaches a very high level. Both the Sorraia and the Przewalski’s horse population have extremely high inbreeding levels (0.21 and 0.38, respectively) due to small effective population sizes and recent severe population bottlenecks [2, 3, 26, 27]. In contrast, the relatively high average inbreeding coefficient (0.156) found for Thoroughbreds in this study is due to many generations of slow inbreeding. A large increase in the rate of inbreeding may lead to more noticeable effects on the reproductive traits of Thoroughbred horses in the future. The prohibition of artificial reproductive technologies (e.g. artificial insemination, cloning and embryo transfer) likely limits the rate of increase in inbreeding in the Thoroughbred population. The use of these technologies in other horse populations has been associated with increases in the rate of inbreeding [28, 29], which could show more measurable effects on reproductive traits. It is also important for Thoroughbred breeders to be vigilant in their selection of sires and dams to avoid overbreeding to successful families as it may increase inbreeding rates in future generations and have unexpected negative effects on the population.

The maternal heritability estimate for gestation length based on our models was 0.562, higher than previous estimates in horses of 0.18–0.39 [12, 30, 31]. This increased heritability estimate may be because all mares in this study have been intensively managed in the same way, minimising the amount of environmental variation that can reduce such estimates. Sires were also estimated to have a smaller, but still significant genetic effect on the gestation length of the foal. Stallions have similarly been found to influence gestation length in other horse breeds [30, 31], and such knowledge could assist in breeding management decisions. For example, avoiding the mating of mares and stallions genetically predisposed to longer gestation lengths, particularly towards the end of the season, could aid in avoiding delayed parturition dates.

Foaling rate had a lower but still significant maternal heritability estimate of 0.058, which similarly to gestation length was slightly higher than previous estimates (0.013–0.024) [32]. Unexpectedly, paternal heritability estimates from our models found no genetic influence of the sire on foaling rate. Only 131 sires were included in this study, so it is possible that a larger sample size would reveal significant heritable effects. Additionally, Thoroughbred stallions that show suboptimal fertility may be gelded and returned to racing. Analyses that include these individuals may reveal more measurable paternal genetic effects on covering success.

In contrast to gestation length and covering success, we found that secondary sex ratio had a negligible maternal heritable component (0.005) with a high standard error, indicating that genetic variation in the mare has no influence on the sex of the foal. However, paternal heritability estimates revealed an extremely small but nonetheless significant effect of sire genetic variation on foal sex ratios (0.011). Other studies in mammals have also found evidence of male-driven sex ratio bias [33, 34]. However, in contrast to these studies, we found no association between the inbreeding level of the sire and offspring sex ratio [33, 34]. It is postulated that higher quality, less inbred fathers produce more male offspring [35]. The artificial breeding practices used in the Thoroughbred population that only allow a small proportion of high-quality males to breed may mitigate any effects of inbreeding on offspring sex ratios, and result only a small measurable heritable effect.

In this study there was found to be little improvement in the estimated breeding value (genetic value) for reproductive traits in Thoroughbred horses from the eighteenth century until present day. The horses for which these values are obtained only include the direct ancestors to the horses with reproductive trait records in our study. Some individuals in previous generations may have had greater variation in genetic value but do not appear in the pedigree of modern Thoroughbreds. We found no change in the mean genetic value for foaling rate until the most recent three generations (Fig. 1). Selection may favour mares that produce many offspring, making them more likely to be present in the pedigree of future generations. On the other hand, female families with low fertility have less chance of appearing in the pedigree of Thoroughbreds in future generations. It is possible that if we had been able to evaluate the genetic value for all horses in the studbook from previous generations, we would have found a greater spread of values. Another reason for the minimal variation in the genetic value of earlier generations may be due to the lack of information conveyed in the binary trait of foaling rate. The near zero values may represent regression to the mean when there is limited information from previous generations.

In the past 30 years (approximately three generations), the average genetic value for foaling rate has decreased, and variation has increased. The decreasing mean may be because horses with a lower genetic value have not yet been selected out of the current Thoroughbred population. These horses may not appear in the pedigree of future Thoroughbreds, such that the values from their generation will show minimal variation. The decreasing average genetic value conflicts with an increased foaling rate reported in recent years [36]. It is likely that the improving fertility rates in the population are due to better management techniques rather than genetic gain. Recent advances in veterinary treatments has led to widespread use of hormonal therapies to increase foaling rate [36]. Increasing commercial demand for mares with good fertility may explain the outlying individuals with high genetic potential in recent years. However, widespread use of intensive veterinary treatments could make selection against individuals with lower genetic value less efficient, resulting in long term reductions in natural reproductive levels of the Thoroughbred population.

Similarly to foaling rate, the genetic value for gestation length has shown little variation since the foundation of the breed (Fig. 4). However, unlike foaling rate, gestation length is not a binary trait. Selection against horses with longer gestation lengths (corresponding to higher genetic values) is likely to occur because they can produce fewer foals throughout their lifetimes. Extremely short gestation lengths may also be selected against because foals born prematurely will have a lower survival rate. These factors will remove individuals with high and low values, resulting in minimal variation and a mean of zero in more distant ancestral generations. It is also possible that the low genetic diversity in previous generations of the pedigree has resulted in reduced variation of the genetic value of these individuals based on breeding records from the current population. In the past 20 years, the average genetic value has reduced, showing that gestation length in Thoroughbred mares has, on average, become shorter. The increase in commercial Thoroughbred breeding during this time may have favoured mares with slightly shorter gestation lengths, as they are more likely to be successfully covered again in the same season. Foals that are born earlier in the season can have a competitive advantage over their peers when racing at a young age, which also favours mares with a decreased gestation length. Variation in genetic values of gestation length has also increased in the recent generations of the pedigree, indicating that there are increasing numbers of mares with genetic potential for very long and short gestation lengths. These individuals may be selected out of the population in future generations.

In contrast to foaling rate and gestation length, the genetic values for racing performance in the Thoroughbred population have increased in recent generations [24]. This may be because Thoroughbred horses are directly selected for good racing success rather than fertility. If racing performance traits are driven primarily by positive selection (selection for advantageous alleles), this would explain the increase in genetic values for these traits over the past few generations. On the other hand, reproductive traits may be driven by mostly negative selection against disadvantageous alleles, explaining the decrease in genetic values of foaling rate in recent generations because there has not been an opportunity for the population to be purged of these genes. Thoroughbred horses have been selectively bred for racing performance since the start of the eighteenth century. However, it is likely that domestic horses have been selected for reproductive traits for many generations prior to the foundation of the Thoroughbred breed. Common signatures of selection for fertility have been found in domestic horse breeds including the Thoroughbred [8, 9]. This could explain why an increase in genetic values for racing performance traits, but not fertility measures, is seen at the foundation of the Thoroughbred breed.

We also found that a number of environmental effects have significant influences on both gestation length and foaling rate. Our results showed that mares foaling down later in the season had significantly shorter gestation lengths (Fig. 5). Our findings agree with previous studies [12, 30, 32] and highlight the importance of photoperiod length for inducing parturition in horses [37]. This pattern may strongly depend on the mare’s location, as photoperiod variation is highly dependent on the proximity to the equator.

We also found that mares who produced male foals had significantly longer gestation lengths than those who produced female foals, possibly because of differences in maternal-foetal hormonal interactions [12, 13, 32, 38]. However, an average difference of 3 days between the gestation lengths of colts and fillies is unlikely to impact breeding management decisions. Gestation length also increased with mare age, which could be explained by changes in hormonal, nutritional and uterine changes as a female ages [13] (Fig. 6). Some studies have found a similar linear increase with age [32], whereas others have found that gestation length is longer in both younger and older mares [12]. This pattern may be dependent on the veterinary management provided to maiden mares. Foaling rate declined with increasing mare age, with mares over the age of 20 having less than 70% success (Fig. 3). Foaling rate reduced dramatically in November and December, most likely due to the accumulation of less fertile mares at the end of the season (Fig. 2). To optimise foaling rate, the breeding of older mares would need to be prioritized because they tend to have longer gestation lengths and lower foaling rates.

In contrast to foaling rate and gestation length, secondary sex ratio was not significantly influenced by any environmental effects included in these models. Mares in a poor nutritional condition at conception have been reported to have an increased chance of successfully carrying a female foetus, with reports of female foal ratios up to 80% [39, 40]. In the Mangalarga Marchador horse breed, higher ratios of female foals were found in older mares [21]. Additionally, increased dam inbreeding in cattle is associated with higher female birth rate [19]. However, we postulate that the high level of veterinary management and care provided to Thoroughbred horses in commercial stud farms examined in this study has resulted in no environmental or inbreeding factors having a measurable influence on foal sex ratios. Differing management of other horse populations (e.g. feed and hormonal supplements) may show more measurable effects on sex ratio. Wild animal populations with more variable environmental conditions and rates of inbreeding may also show different trends.

## Conclusions

In this study we found that inbreeding had no measureable effect on reproductive traits in Australian Thoroughbred horses. Although this contrasts with previous findings in racing performance traits, we postulate that inbreeding levels are not yet high enough to have a measurable effect on the Thoroughbred reproduction traits examined in the current study. The effects of artificial reproductive technologies on natural fertility rates in other horse populations should be examined to ensure that such practices do not result in long-term reductions in natural fertility levels. Genomic scans for reproduction traits in Thoroughbred and other breeds may assist in understanding genetic variation that influences fertility. We also found that unlike racing performance, there has been little increase in the breeding value of reproductive traits in Thoroughbred horses. Breeding values of foaling rates have decreased in recent generations, possibly because these traits are primarily governed by negative rather than positive selection. Further monitoring of these traits in future generations would assist in understanding the selective forces influencing these traits.

## Methods

Reproductive trait data were provided by three large Australian Thoroughbred stud farms that provide a representative sample of the population as a whole. These data included the mating records of 12,922 mares bred to 131 stallions between 2000 and 2017. The scan status of each mare covering (negative or positive at the first scan 15 days after covering) was transformed into a binary trait. The sex of each live foal recorded in the dataset (*n* = 7578) was also transformed into a binary trait (female = 1, male = 0). Additionally, more detailed reproductive trait data were available for 152 mares mated to 89 stallions over multiple seasons (*n* = 764 foals), including the date of the foal birth. The gestation length of each live foal birth was calculated from these data.

The pedigree for all the mares, stallions and conceptuses included in the study dating back to the founders of the population consisted of 92,852 records. We used the CFC program (version 1) to reorder the pedigree to ensure that each individual was listed after their parents and to estimate Wright’s inbreeding coefficient for all individuals [41]. We also used CFC to estimate the overall average of the average number of generations in the pedigree for each mare and stallion [41].

The genetic and environmental influences on the foaling rate, gestation length and sex ratio were estimated in ASReml-R [42], using a linear mixed model for gestation length, and a generalised liner mixed model for the binary traits of foaling rate and foal sex. Details of the fixed and random effects in each model are included in Table 1. The outcome variables were foaling rate, the sex of the foal and the gestation length. The fixed effects included in each model were: inbreeding coefficient (of the mare, the stallion and the foal), the month of covering, the year of covering, the age of the mare and the stud farm. An animal model was implemented through an inverse relationship matrix using the pedigree in ASReml-R [42]. Multiple coverings of the same mare in the same season were accounted for in the models by inclusion of a permanent environment effect of the mare, as a random effect.

**Table 1:** Mixed models of each fertility trait for Australian Thoroughbred horses. Foal, sire and dam inbreeding were estimated in separate models. Model descriptions are in R syntax, not full mathematical notation

Outcome variable	Model type	Models
**Gestation length**	LMM	GL ~ mare.age + month + foal.sex + season + *F* + ped (mare) + ide (mare) GL ~ mare.age + month + foal.sex + season + *F* + ped (sire) + ide (sire)
**Foaling rate**	Binomial GLMM	CS ~ mare.age + month + season + *F* + ped (mare) + ide (mare) CS ~ mare.age + month + season + *F* + ped (sire) + ide (sire)
**Foal sex ratio**	Binomial GLMM	SR ~ mare.age + month + season + *F* + ped (mare) + ide (mare) SR ~ mare.age + month + season + *F* + ped (sire) + ide (sire)

*LMM* Linear mixed model, *GLMM* Generalised linear mixed model, *GL* Gestation length, *CS* Foaling rate, *SR* Foal sex ratio, mare.age = age of the mare at the time of covering, month = month of covering, *F* = inbreeding coefficient of the sire, dam or foal (models were run separately for each), ped = pedigree of the mare, ide = permanent environmental effect of the mare

The heritability of each outcome variable was estimated using the variance component estimates of the fitted models. Significance of model terms was evaluated through Wald statistics and the estimated value of each fixed effect tabulated. Estimated breeding values (i.e. genetic values) for foaling rate and gestation length were calculated as best linear unbiased predictions for each individual in the pedigree using the models fitted in ASReml-R. This method uses the available phenotypic data and the associated pedigree structure in the models to provide genetic value estimates for all animals in the pedigree.

## Data Availability

The data that support the findings of this study are available upon reasonable request but restrictions apply to the availability of these data, which were used under license for the current study, and so are not publicly available.
